# When MED16 Meets Plant Growth, Development, and Stress Response

**DOI:** 10.3390/ijms27052475

**Published:** 2026-03-07

**Authors:** Luyi Li, Shu-Li Qi, Chunxiu Shen, Tian-Tian Zhi, Jie Zou, Gang Chen

**Affiliations:** 1 Jiangxi Key Laboratory of Crop Growth and Development Regulation, College of Life Sciences and Resources and Environment, Yichun University, Yichun 336000, Chinashenchunxiu@126.com (C.S.); 190055@jxycu.edu.cn (T.-T.Z.); jetzou@126.com (J.Z.); 2 School of Life Sciences, University of Warwick, Coventry CV4 7AL, UK

**Keywords:** MED16, Mediator complex, abiotic stress response, growth–defence trade-off, transcriptional regulation

## Abstract

Mediator is a central transcriptional coactivator that connects sequence-specific transcription factors with RNA polymerase II to control inducible gene expression in plants. MED16 is a Mediator tail module subunit that functions as a context-dependent integrator, helping coordinate developmental programs with environmental adaptation. This review summarizes current evidence for MED16 function from structural and evolutionary perspectives to physiological outputs, with emphasis on how MED16 interacts with transcription factors and other Mediator subunits to shape RNA polymerase II engagement at target loci. In terms of development, MED16 contributes to organ growth and root system architecture, and comparative studies have revealed that it plays conserved roles in lineage-specific wiring. Under abiotic stress, MED16 supports the efficient activation of stress-inducible transcription, including cold acclimation and nutrient stress responses such as phosphate starvation-dependent root remodeling. In immunity, MED16 modulates salicylic acid- and jasmonate/ethylene-associated defence outputs and can be targeted by plant viruses, which is consistent with its role in antiviral transcriptional responses. Mechanistically, MED16 participates in cooperative and competitive interactions within the Mediator complex that tune hormone-responsive outputs, exemplified by MED25-related competition in abscisic acid signalling. We highlight key limitations and future directions, including the need for mechanistic validation beyond *Arabidopsis*, clearer models of dosage control in crops, improved understanding of context-dependent tail configurations, and high-resolution mapping of MED16 interaction interfaces.

## 1. Introduction

Mediator is a core coactivator in eukaryotic transcription that links gene-specific transcription factors with RNA polymerase II (Pol II) to regulate gene expression [1,2,3]. In plants, Mediator comprises ~30–35 subunits that are typically organized into four modules: head, middle, tail, and kinase [1,2,3]. The head and middle modules form a core scaffold that is associated with Pol II, whereas the tail module provides a major interface for DNA-bound transcriptional activators [1,4,5,6]. Structural and functional studies further suggest that changes in tail-associated contacts can be coupled to changes in the core complex and Pol II engagement [1,4,5,6]. Within this framework, MED16 is a tail module subunit, and evidence links it to Pol II-dependent transcriptional control, providing a plausible route by which upstream signals are converted into gene expression outputs [1,7].

Work in *A. thaliana* connects MED16 to diverse stress and developmental phenotypes. Under cold conditions, MED16 is required for cold acclimation and supports Mediator and Pol II recruitment at *CBF*-responsive loci [8,9]. MED16 also contributes to nutritional stress responses, including iron and phosphate limitation, and it participates in salicylic acid- and jasmonate/ethylene-associated defence programs [7,10,11]. In development, MED16 affects endoreplication and organ growth through *CCS52A1/CCS52A2*-linked regulation [12,13]. In contrast, mechanistic evidence in cereal crops is still limited. Current reports indicate that *OsMED16* influences rice growth, lesion-like cell death, and defence-related transcription and that *circZmMED16* is associated with maize development [14,15].

These observations motivate two testable, mechanistic questions. One concerns how MED16 can couple distinct upstream pathways to a shared Pol II-dependent output and whether recurring steps in Mediator–Pol II engagement can be identified across cold, nutrient, immunity, and growth contexts [1,7,8,9,10,11,12,13]. The other concerns scope: to what extent do the Arabidopsis-derived models hold in cereals, where functional and mechanistic data remain sparse [14,15]? We therefore set three linked tasks for this review: we consolidate the evidence for MED16 function across major physiological contexts; we critically assess the strength and limits of proposed mechanistic models in terms of Mediator/Pol II recruitment and promoter-level control; and we map the most important gaps that block generalization across species and pathways [7,8,9,10,11,12,13,14,15].

### Structure and Cross-Species Functions of MED16

MED16 is a subunit of the Mediator tail module [1,13,16]. The cryo-EM structure of fungal Mediator places MED16 near the center of the tail and shows a clear two-domain organization, with an N-terminal WD40 domain and a C-terminal helical domain [1]. In this structure, the WD40 domain contacts MED5, and the helical domain contacts MED15, which puts MED16 at a key interface inside the tail [1]. The same work also revealed that the C-terminal region of MED14 contacts both MED15 and the C-terminal region of MED16; thus, MED16 sits close to the MED14–tail connection that links the tail to the core scaffold [1]. Although Mediator subunits show low sequence similarity across species, secondary structure prediction suggests that MED16 maintains similar domain features between *C. thermophilum* and *S. cerevisiae*, which supports structural conservation at the fold level [1]. This structural layout also fits a simple model in which tail rearrangement can be communicated through MED14 and tail interfaces, which helps explain how activator-facing surfaces can be coupled to Pol II engagement [1,17,18,19].

These plant data support that MED16 is conserved, but they also show species-specific differences in function. In rice, *OsMED16* shows substantial sequence identity to *AtMED16*, and OsMED16 is a nuclear protein with broad expression across organs, which is consistent with its role as a core transcriptional regulator [14]. Genetic and dosage perturbations in rice indicate that *OsMED16* is required for normal growth. CRISPR/Cas9 disruption caused severe defects and seedling lethality, and overexpression inhibited growth, resulting in lesion-like cell death, and triggered reactive oxygen species accumulation and early senescence [14]. *OsMED16* overexpression also reduced yield, which suggests that OsMED16 activity needs tight control in planta [14].

This work in maize adds another layer because the *MED16* locus can also act through a circular RNA. *circZmMED16* originates from an exon of the *ZmMED16* gene, is enriched in the cytoplasm, and delays flowering when it is overexpressed in both maize and Arabidopsis [15]. Mechanistic assays revealed that circZmMED16 forms an RNA/RNA duplex with ZmAPS1 mRNA in vivo, which is linked to reduced ZmAPS1 RNA and protein levels, lower AGPase activity, and lower starch levels [15]. In the same study, ZmMED16 RNA itself did not show the same interaction pattern as ZmAPS1 in the reported assays, which suggests that this effect is specific to the circular RNA output from the locus [15].

With these structural and cross-species observations in place, the next section focuses on how MED16 connects defined upstream inputs, such as cold and nutrient signals and hormone-linked immunity, to changes in Pol II-dependent transcription in plants.

## 2. Regulatory Functions of MED16 in Core Developmental Processes

### 2.1. Vegetative Growth: Regulation from Cell to Organ

#### 2.1.1. Brakes on the Cell Cycle and Endoreplication: Size Control in Leaf and Petal Development

Endoreplication is a common route to increase cell size during plant organ growth, and it is controlled by cell cycle regulators that shift cells from the mitotic cycle to the endocrine cycle [20,21]. A key step in this shift is APC/C activation by CCS52 proteins, and CCS52A1 and CCS52A2 act as major regulators of endocycle entry and progression [20,21,22,23]. This pathway is also linked to E2F-related transcriptional control, and DEL1 represses endoreduplication in *Arabidopsis* [20,22,24].

**Figure 1:**
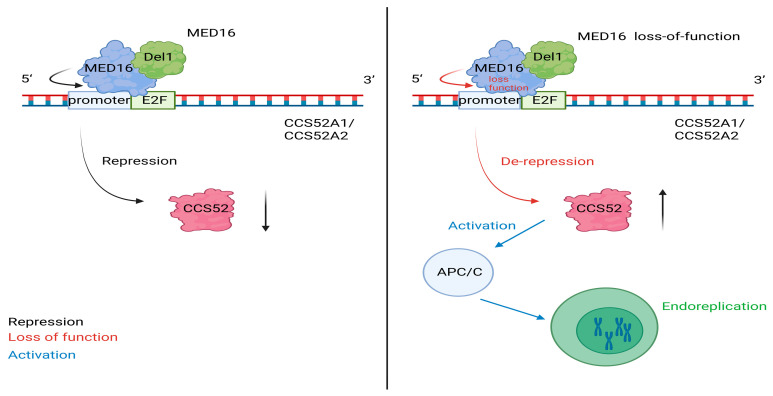
MED16–DEL1 represses CCS52 to limit endoreplication during organ growth. (**Left**) (MED16): MED16 cooperates with the transcriptional repressor DEL1 at E2F-containing promoter regions to repress *CCS52A1/CCS52A2* expression, which limits CCS52 abundance and restrains APC/C activity, thereby restricting endoreplication. (**Right**) (MED16 loss-of-function): Loss of MED16 causes derepression of *CCS52A1/CCS52A2*, leading to increased CCS52 levels and enhanced APC/C activation, which promotes endoreplication and increases nuclear ploidy. Created in BioRender. Li, L. (2026) https://BioRender.com/1kh95b8 (accessed on 25 February 2026).

Within this framework, MED16 acts as a brake on endoreplication and cell growth in the aerial organs of *A. thaliana*. Loss-of-function *MED16* mutants present enlarged leaves and petals, and this phenotype is linked to increased cell size and altered growth during the early primordium stages [12] (Figure 1). Cytological data and flow cytometry profiles revealed increased nuclear ploidy in *MED16* tissues, with increased proportions of 16C and 32C nuclei and the appearance of 64C nuclei that were not detected in the wild type [12].

At the transcriptional level, MED16 represses *CCS52A1* and *CCS52A2* [12]. These genes encode APC/C activators that promote endocycle progression [20,21]. Chromatin immunoprecipitation results suggest that MED16 is associated with the promoter regions of *CCS52A1* and *CCS52A2*, which contain E2F-related cis-elements, and that the transcript levels of both genes are relatively high in the *MED16* background [12]. MED16 also interacts with the transcriptional repressor DEL1 (DP-E2F-like 1), and this interaction is linked to the repression of *CCS52A2*, which is consistent with prior work indicating that DEL1 is involved in endocycle control [12,22,24]. In the same study, DEL1 does not explain *CCS52A1* regulation, which suggests that MED16 represses *CCS52A1* through other partner factors [12].

Genetic tests further support that *CCS52A1* and *CCS52A2* contribute to the MED16 phenotype. Mutations in *CCS52A2* and *CCS52A1* suppress cell enlargement and high ploidy accumulation in MED16, but this suppression is not complete, which suggests additional routes that act with this axis [12]. The next subsection moves from this leaf and petal growth mechanism to how MED16 shapes root architecture by altering auxin transport and auxin response gene expression.

#### 2.1.2. Shaper of Root Architecture: A Hub for Auxin Signal Integration

MED16 has been reported to control root development by influencing auxin transport and signalling in *Arabidopsis thaliana* [25]. In that study, med16 mutants presented reduced PIN1 signalling in the root meristem and lower expression of the auxin signalling repressors IAA14 and IAA28, indicating that MED16 is required to maintain normal auxin transport and signalling levels [25]. Taken together, these observations support an ordered view in which altered PIN1-associated auxin distribution and reduced Aux/IAA repression converge on transcriptional outputs, placing MED16 at the step that links upstream signalling status to Pol II-dependent promoter activity. Direct evidence defining promoter features or chromatin states that confer locus specificity remains limited, and this is a clear evidence gap for auxin-related root phenotypes. Such specificity could arise from promoter cis-element composition, local accessibility, or context-dependent Mediator interactions, and it can be tested by promoter-resolved Pol II/Mediator occupancy together with chromatin accessibility profiling in relevant genotypes.

This coordination may represent a buffering role that balances auxin redistribution and the transcriptional response. NPA, an auxin transport inhibitor, directly binds to PIN proteins and blocks efflux activity, disturbing the auxin gradient [26,27]. In wild-type plants, NPA strongly suppresses primary root elongation, whereas med16 mutants show reduced sensitivity and retain elongation in the elongation zone, accompanied by increased lateral root formation and biomass under treatment [25]. This differential NPA response is consistent with a shift in auxin responsiveness when MED16 function is impaired. Consistently, reduced IAA14 and IAA28 expression in MED16 may keep downstream ARF7/ARF19-LBD modules more active during lateral root development [25,28]. Together, these data support a working model in which MED16 helps define auxin sensitivity by coordinating PIN-mediated transport with Aux/IAA-dependent signalling repression, while the promoter-level basis of target selectivity remains unresolved.

**Figure 2:**
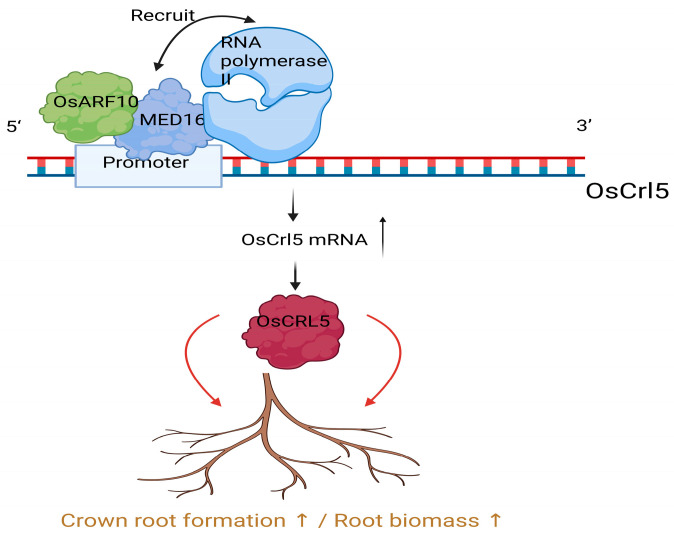
OsMED16–OsARF10 promotes *OsCrl5* transcription to support crown root formation and root biomass. OsARF10 binds the OsCrl5 promoter and interacts with the Mediator tail subunit OsMED16. OsMED16 facilitates RNA polymerase II recruitment to the *OsCrl5* promoter, leading to increased *OsCrl5* mRNA accumulation and elevated OsCRL5 protein levels. Upregulated OsCRL5 promotes crown root formation and increases root biomass. Created in BioRender. Li, L. (2026) https://BioRender.com/hdl8if9 (accessed on 25 February 2026).

Evidence from monocots has indicated a conserved connection between MED16 and auxin-regulated root architecture. In rice, OsMED16 promotes crown-root formation and root biomass, and loss-of-function mutants present reduced crown-root number and weight with lower yield [29]. Mechanistically, OsMED16 interacts with OsARF10, which binds and activates the promoter of *OsCrl5*, and OsMED16 facilitates RNA-pol II recruitment to this promoter [29]. This shared MED16-ARF mechanism underlies conserved auxin transcription coupling, although specific ARFs and targets differ between species, reflecting distinct root architectures [25,29] (Figure 2).

### 2.2. Growth–Defence Trade-Off: Dosage-Dependent Precise Regulation

**Figure 3:**
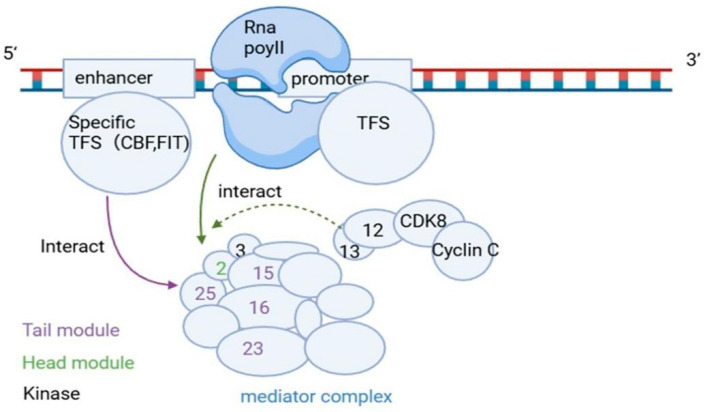
Model of MED16-mediated transcriptional activation in plant abiotic stress responses. Gene-specific transcription factors (e.g., CBF and FIT) bind DNA near enhancers or promoters and interact with MED16 in the Mediator tail module. This connection helps recruit and assemble the Mediator complex at the promoter, supporting RNA polymerase II (Pol II) engagement and transcription initiation. The figure also indicates the kinase module (e.g., CDK8–Cyclin C) as a dynamic component that can modulate transcription output. Created in BioRender. Li, L. (2026) https://BioRender.com/zw93e78 (accessed on 13 February 2026).

OsMED16 shows strong dosage sensitivity in rice: both loss of function and overexpression are associated with major growth defects. Jiang et al. reported that OsMED16 knockout mutants are seedling lethal, whereas *OsMED16* overexpression causes growth retardation accompanied by lesion-like cell death, reactive oxygen species accumulation, and constitutive upregulation of defence genes such as *OsPR1a/OsPR1b* and *OsPR10a* [14]. These findings suggest that the abundance of OsMED16 requires tight control and that increased OsMED16 activity can be coupled to defence-associated transcription alongside growth inhibition [14] (Figure 3).

A related view emerges from *Arabidopsis*, where genetic interactions among tail module subunits indicate that tail module imbalance can reprogram growth-linked and stress-linked outputs. Dolan et al. reported that the semidominant *MED5* allele *REF4-3* causes dwarfism with broad transcriptional reprogramming, whereas the *med16* (*sfr6-2*) mutation suppresses *REF4-3*-associated growth inhibition and many *REF4-3*-linked expression changes [13]. In the same study, transcriptome analyses linked MED16 perturbation to shifts in genes annotated for stress and defence responses, and the authors reported that the expression of stress-response genes can change in opposite directions in the *REF4-3* and *MED5ab* backgrounds [13]. They also reported that the suppression of *REF4-3* dwarfism by MED16 does not fully restore the associated metabolic phenotype, suggesting that growth outputs and stress-linked metabolic outputs can be partly uncoupled in this tail module context [13].

Together, these dosage- and context-dependent effects provide a framework for the next section, which examines how MED16 contributes to defined abiotic and biotic stress response transcription programs in plants.

## 3. Pivotal Role of MED16 in Biotic and Abiotic Stress Responses

### 3.1. Abiotic Stress: From Perception to Gene Activation

#### 3.1.1. Cold Stress: An Essential Coactivator of the CBF Pathway

AP2-type CBF transcription factors (CBF1, CBF2, and CBF3) mediate cold acclimation in *Arabidopsis thaliana* by activating *COR* (cold-regulated) genes through binding CRT/DRE motifs in *COR* promoters, which supports freezing tolerance [30,31]. Cold induces CBF expression, and the output of CBF is also shaped by the posttranslational control of upstream regulators such as Inducer of CBF Expression 1 (ICE1), including SUMOylation- and ubiquitination-linked regulators [30,31,32].

Genetic analysis revealed that SFR6 functions downstream of the CBF translation step. In *sfr6* mutants, the levels of *CBF* transcripts and proteins are similar to those in the wild type, but the expression of CBF-regulated *COR* genes is not induced, even when CBFs are overexpressed [8]. *SFR6* encodes the Mediator subunit MED16, a nuclear factor required for cold-responsive transcription [9].

In this context, MED16 does not control CBF recruitment to tested target promoters and is not required for the acetylation of the cold-associated histone H3 at the loci examined [9]. Instead, the main defect is linked to the recruitment of Mediator and RNA polymerase II (Pol II) to CBF-regulated cold-inducible genes such as *KIN2* and *GOLS3* [9]. Under low temperature, Pol II occupancy at the transcription start sites of *KIN2* and *GOLS3* is greater in the wild type than in *SFR6*, and the defect in *SFR6* is strongest at the initial recruitment step, with no clear effect on transcriptional elongation in the reported assays [9].

MED16 also acts with other Mediator subunits, including MED2 and MED14, to support Mediator and Pol II recruitment, and loss of these subunits reduces *COR* gene induction and weakens cold acclimation, resulting in phenotypes such as increased electrolyte leakage and reduced survival after freezing [8,9]. MED16 is also required for the expression of a subset of cold-inducible genes that contain EE motifs [9] (Figure 3).

#### 3.1.2. Nutritional Stress: A Regulator of Iron and Phosphate Homeostasis

Under Fe deficiency, genetic evidence has shown that MED16 (also called YID1) and MED25 participate in the transcriptional control of the *FIT*-based iron uptake program, including *FIT* and its downstream genes *IRT1* and *FRO2* [10]. MED25 interacts with EIN3/EIL1 so that ethylene signalling can affect *FIT*-related transcription under iron limitation [10].

Under Pi starvation, MED16 is needed for a large part of the low-Pi transcriptional response in roots, and it works in the *STOP1-ALMT1* regulatory axis [11,32]. At low Pi, MED16 loss-of-function mutants have longer primary roots with sustained meristem activity, and they have reduced lateral root formation; thus, MED16 is needed for the typical low-Pi root architecture program [11]. RNA-seq results also revealed that most low-Pi-responsive genes in the root apex depend on MED16 (approximately 85% are affected in MED16-2), and ALMT1 is expressed at relatively low levels and is relatively weakly activated in MED16 [11].

MED16 is also associated with STOP1, and it is needed to activate STOP1 targets such as ALMT1, which promotes malate release from roots [11]. Malate efflux supports apoplastic Fe mobilization and redox changes, triggering CLE14 signalling and inhibiting root meristem activity, which results in the formation of a malate-Fe-CLE14 checkpoint module under Pi limitation [11,33,34]. This finding is also supported by rescue tests, since the addition of malate or the CLE14 peptide restored the low-Pi short-root and meristem exhaustion phenotype in med16 mutants; thus, MED16 acts upstream in this pathway [11].

### 3.2. Biotic Stress: A Convergence Point for Immune Signalling Pathways

Salicylic acid (SA), together with its major transcriptional coactivator NPR1, controls systemic acquired resistance (SAR) [7]. NPR1 interacts with TGA transcription factors and activates SA-responsive *PR* genes, including *PR1*, *PR2*, and *PR5* [7,35]. In parallel, the jasmonic acid/ethylene (JA/ET) pathway regulates defence against necrotrophic pathogens such as *Botrytis cinerea* and *Alternaria brassicicola*, and marker genes include *PDF1.2*, *CHIB*, and *HEL* [7,36,37]. Because SA- and JA/ET-associated outputs can be coordinated or opposed depending on context, MED16 provides a useful entry point to discuss how mediators contribute to immune transcription across pathways.

#### 3.2.1. SA/SAR Signalling: MED16 in Systemic Acquired Resistance

Genetic and molecular evidence places MED16 downstream of SA in the SAR pathway, since *MED16* mutants show strongly reduced induction of *PR1*, *PR2*, and *PR5* after SA/BTH-related treatments [7]. MED16 also affects NPR1 accumulation, with reduced NPR1 protein levels and lower NPR1 transcript levels reported in med16/sfr6 under specific conditions [7]. In addition, in pathogen-induced expression programs, MED16 suppresses the expression of SAR-negative regulators such as WRKY38 and WRKY62 and promotes the expression of SAR-positive regulators such as AZI1 and DIR1. Together, these findings support a role for MED16 in shaping SA-associated immune transcription and provide a framework for understanding MED16 function when SA signalling intersects with other defence pathways.

#### 3.2.2. JA/ET Signalling: Specific Coactivation Against Necrotrophic Pathogens

MED16 is a tail module subunit of Mediator and contributes to basal resistance against the necrotrophic fungus *Sclerotinia sclerotiorum* in *Arabidopsis thaliana* [38,39]. Current evidence supports a stepwise model in which JA/ET inputs are converted into Pol II-dependent transcription through a WRKY33–MED16 axis. MED16 is associated with WRKY33 in yeast and in planta, and this association is linked to the activation of the JA/ET defence genes *PDF1.2* and *ORA59* [38]. After MeJA plus ACC treatment, chromatin immunoprecipitation assays revealed that MED16 is required for efficient recruitment of RNA polymerase II to the coding regions of *PDF1.2* and *ORA59* and that both Pol II recruitment and gene induction are strongly reduced when MED16 function is compromised [38]. This requirement is branch-specific within JA signalling: MED16 is necessary for the JA/ET branch that induces *PDF1.2/ORA59*, but it is dispensable for the JA wound branch that activates *VSP1*, *VSP2*, and *JR1* [38]. Consistent with distinct transcription factor usage, WRKY33 works with MED16 in the JA/ET branch, whereas MYC2 is a major activator of wound-responsive transcription [38,40]. Accordingly, the WRKY33-dependent induction of *PDF1.2/ORA59* and the *WRKY33* overexpression-associated resistance phenotype against *S. sclerotiorum* require MED16 [38].

Although the transcriptional control step is supported, several downstream links that connect altered gene expression to defence outputs are not explicit in the available evidence summarized here. In particular, it remains unclear which induced products are most important for restricting necrotroph growth in this context, whether their abundance or localization is altered, and how these changes translate into measurable cellular readouts, such as ROS dynamics, cell wall-associated reinforcement, or shifts in primary/secondary metabolism. Where these steps have not been tested, they represent concrete gaps that matter for the mechanism because they define how a MED16-dependent Pol II recruitment event is converted into tissue-level resistance. Crop relevance in oilseed rape (*Brassica napus*). Sclerotinia stem rot, caused by *S. sclerotiorum*, is a major disease constraint for oilseed rape [41,42]. In *B. napus*, *BnMED16* transcript levels increase upon infection, and constitutive overexpression of *BnMED16* in a susceptible background improves resistance in inoculation assays [41]. This enhanced resistance is correlated with stronger activation of defence programs, including ROS-related responses, defence gene induction, and the reinforcement of cell wall-associated defences [41]. Evidence of protein interactions further supports a signalling-to-transcription framework in which BnMED16 associates with BnWRKY33 and BnMED25, whereas BnMED25 associates with JA/ET components such as MYC2, COI1, and EIN3 [41]. Together, these findings support a working model in which MED16 helps couple *WRKY33*-centered JA/ET transcriptional outputs to crop resistance [41]. However, the mechanistic chain beyond transcription remains insufficiently resolved in the crop setting, as the identity of the key defence products downstream of these transcriptional changes, their site of action, and the quantitative links to cell wall composition or metabolic outputs are not yet clearly defined. Addressing these steps would strengthen causal inference and would also help distinguish whether MED16 primarily amplifies transcription broadly or preferentially channels JA/ET outputs toward particular effector classes relevant to necrotroph defence.

#### 3.2.3. Viral Defence: A Critical Target of Pathogen Attack

During turnip mosaic virus (TuMV) infection, the viral NIa-Pro protease cleaves MED16, which disrupts its nuclear localization and weakens JA/ET-dependent defence responses, thereby promoting viral accumulation [41]. NIa-Pro cleaves MED16 at a specific site, and this cleavage removes the nuclear localization signal, which is consistent with reduced nuclear accumulation and reduced Mediator-dependent activation of defence gene transcription [43]. This mode of action links NIa-Pro activity to compromised MED16-dependent defence gene regulation in the JA/ET module, and it affects downstream defence outputs associated with JA/ET signalling [43]. Compared with wild-type plants, *med16* mutants consistently exhibit greater TuMV infection efficiency and improved aphid vector performance, which supports the role of MED16 in defence against both the virus and its insect vector [43].

## 4. Molecular Mechanisms of MED16 Function: Interactions, Competition, and Regulation

### 4.1. Dynamic Interaction Networks with Key Transcription Factors

Genetic and molecular studies have shown that MED16 regulates transcription by forming context-dependent interaction modules with distinct transcription factors, which link pathway inputs to Mediator and RNA polymerase II (Pol II) recruitment at target genes [7,9,12,38]. These modules have been supported by protein-protein interaction evidence (for example, yeast-based assays and in planta interaction tests) and by chromatin-based evidence that tracks Mediator/Pol II occupancy at target loci [7,9,12]. During vegetative growth, MED16 binds the transcriptional repressor DEL1 and represses *CCS52A2*, which affects endoreduplication and cell expansion [12]. In hormone signalling, MED16 modulates ABA responses by binding to ABI5 and affecting ABI5-mediated transcriptional outputs [16]. In auxin signalling, *ARF*-dependent transcriptional programs are linked to Mediator function and Pol II-associated transcriptional control at target promoters, providing a route for auxin-responsive gene regulation [44]. During nutrient stress, MED16 contributes to iron homeostasis through the MED25-EIN3/EIL1-FIT-associated module [10], and it supports phosphate starvation responses through the *STOP1-ALMT1* axis [11]. In immune signalling, MED16 cooperates with *WRKY33* to support JA/ET defence gene activation during necrotrophic infection [38], and it also supports SA-dependent SAR by shaping the *NPR1* regulatory network [7]. Under cold conditions, MED16 contributes to the recruitment of Mediator and Pol II to CBF-responsive, cold-regulated genes, which supports cold-induced transcription [9]. In these contexts, a recurring feature is that MED16 helps couple pathway-specific transcription factors to productive transcriptional outputs by shaping Mediator/Pol II recruitment or activity at defence- and stress-responsive genes [7,9,12]

Table 1 summarizes the transcription factors that directly interact with MED16. In the next subsection, we focus on hormone-specific fine-tuning and outline how MED16 helps shape ABA-dependent transcription, including competition-based control of ABI5-regulated targets [16].

### 4.2. Fine-Tuning of Hormone Signalling Pathways: A Competitive Regulatory Model Understood by ABA

#### ABA Signalling: Interaction with ABI5, Competitive Binding with MED25, and Positive Regulation of the ABA Response

Abscisic acid (ABA) is a plant hormone that is rapidly induced by abiotic stresses, such as drought, high salinity, and low temperature, and it promotes stress survival and controls seed maturation and dormancy. It also affects seed germination, root growth, senescence, and stomatal movement [45,46]. ABI5 is a basic leucine zipper (bZIP) transcription factor in the ABA pathway, and it shares conserved motifs with ABF/AREB factors in the same clade [47]. ABI5 activity is directly regulated by ABA signalling because ABI5 and ABF/AREB proteins can be phosphorylated by SnRK2 kinases at conserved RXXS/T sites in an ABA-dependent manner [48,49,50]. Yeast one-hybrid assays also revealed that ABI5 can bind the promoter regions of genes encoding ABA coreceptors, including ABI1 and ABI2, which supports a feedback link between ABI5-driven transcription and the output of ABA signalling [48].

MED16 is a Mediator tail subunit that acts as a positive regulator of ABA responses in *Arabidopsis* [16]. Loss of MED16 reduces ABA sensitivity during seed germination and early seedling growth, and the results of transcriptome data show that ABA-induced reprogramming is weakened in *med16*, with lower induction of ABA-responsive genes such as *RD29A*, *RD29B*, *COR15A*, and *COR47* [16]. At the molecular level, MED16 interacts with ABI5, and MED16 and MED25 can competitively bind ABI5, which links subunit competition to opposite mutant phenotypes in the ABA response [16]. ChIP assays further revealed that ABA promotes the recruitment of MED16 to ABI5 target promoters, including EM1 and EM6, which connects MED16-ABI5 binding to promoter-level control of ABA-responsive transcription [16].

### 4.3. Posttranslational Modifications and Regulation of Protein Stability

Posttranslational modifications can change the stability of key signalling proteins, which allows fast and precise control of cellular responses [51,52]. Ubiquitination is one main way to control steady-state protein levels, and it can also regulate Mediator subunits [52,53,54]. In jasmonate signalling, MED25 is targeted for degradation by two homologous E3 ubiquitin ligases, MBR1 and MBR2 [54,55]. These RING H2-type ligases interact with the vWF-A domain of MED25 and promote its ubiquitination-linked proteasome degradation [52,53,54,56]. MED16 can counter this process by binding competitively to the same vWF-A domain, which blocks MBR1/2-dependent ubiquitination and degradation of MED25 [55]. This MED16-MBR1/2 competition module helps control MED25 homeostasis and can tune the amplitude of JA-dependent transcriptional outputs [55]. Because MED25 also functions in other pathways, changes in MED25 stability may also affect MED25-dependent responses beyond those associated with JA signalling, including iron homeostasis modules [10,55].

Pathogens can also intervene at the protein level through proteolytic cleavage. During turnip mosaic virus infection, the viral nuclear inclusion protease (NIa-Pro) cleaves MED16, which weakens defence gene activation and supports viral infection [43]. In addition, other posttranslational modifications, such as phosphorylation, may further alter the activity or stability of MED16 and related Mediator components [51,55,57]. Decoding these protein-level controls helps explain how Mediator function is tuned across development and stress responses [55].

### 4.4. Beyond the Coding Gene: Novel Regulatory Layers Derived from Noncoding RNA

Recent findings suggest that the *MED16* locus can generate functional circular RNAs, which add a noncoding RNA layer to MED16-linked developmental regulation [15]. In maize, Tang et al. identified a circular RNA derived from exon 8 of *ZmMED16* through reverse splicing [15,58]. *circZmMED16* shows typical circular RNA features, including resistance to RNase R digestion, lack of a poly(A) tail, and predominant cytoplasmic localization [15,59,60]. Expression profiling further revealed strong accumulation in reproductive tissues, which supports a role in floral transition [15].

**Figure 4:**
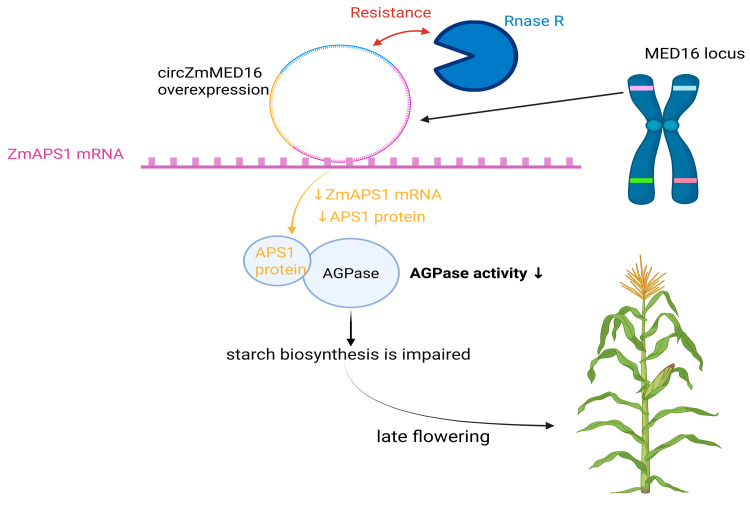
Proposed model for *circZmMED16*-mediated repression of the ZmAPS1–AGPase module leading to impaired starch biosynthesis and late flowering. The *ZmMED16* locus generates a circular RNA (circZmMED16) that displays RNase R resistance. Upon *circZmMED16* overexpression, *circZmMED16* binds to *ZmAPS1* mRNA, resulting in reduced *ZmAPS1* mRNA abundance and decreased APS1 protein accumulation. Because APS1 encodes a small subunit of ADP-glucose pyrophosphorylase (AGPase), a reduction in APS1 expression is associated with decreased AGPase activity, which compromises starch biosynthesis and ultimately contributes to a late-flowering phenotype. Created in BioRender. Li, L. (2026) https://BioRender.com/hdl8if9 (accessed on 25 February 2026).

These functional analyses indicate that *circZmMED16* negatively regulates flowering. Transgenic maize and Arabidopsis lines overexpressing *circZmMED16* flower late, whereas manipulation of the linear *ZmMED16* transcript does not reproduce this phenotype, which supports an RNA-specific effect rather than a MED16 protein-coding effect [15]. Consistently, *circZmMED16* overexpression reduces the leaf starch content, which suggests that altered carbohydrate metabolism is a physiological basis for late flowering [15]. Mechanistically, circZmMED16 directly binds ZmAPS1 mRNA, which encodes the small subunit of ADP-glucose pyrophosphorylase (AGPase), a key control point in starch biosynthesis [15,61,62,63]. RNA pull-down and RNA immunoprecipitation assays confirmed the circZmMED16-ZmAPS1 interaction, and *circZmMED16* overexpression reduced both *ZmAPS1* transcript abundance and protein accumulation, which decreased AGPase activity [15]. As a result, starch biosynthesis is impaired, and upstream metabolites, including glucose-6-phosphate (G6P), are proposed to accumulate in *circZmMED16*-overexpressing plants [15]. These metabolic changes provide a mechanistic link between circZmMED16 activity and delayed flowering, which is consistent with the established role of carbohydrate status in controlling floral transition [64,65,66,67] (Figure 4).

More broadly, this maize study highlights that Mediator subunit loci can contribute regulatory outputs not only through protein coding but also through noncoding RNA products [15]. Given that circular RNAs derived from protein-coding genes are prevalent in plants, related RNA-based regulatory modes may exist for Mediator subunits in other taxa, even though direct evidence beyond circZmMED16 remains limited [59,60,68,69].

## 5. Conclusion and Future Perspectives

### 5.1. MED16: A Central Hub in Plant Adaptive Regulation

MED16 is a key subunit of the Mediator tail module and is linked to transcriptional control, which helps balance growth with stress responses. Evidence across genetics and transcriptomics suggests that MED16 acts as an integrator rather than a component of a single linear pathway. During development, MED16 contributes to transcriptional programs that affect endoreduplication, cell expansion, and organ growth, and it is also connected to auxin-related root architecture. Studies across species indicate a conserved role of MED16, whereas specific outputs may differ between dicots and monocots.

MED16 is also associated with responses to cold and nutrient limitation, and it supports stress-induced transcription by facilitating Mediator and RNA polymerase II activity at target genes. In immunity, MED16 is linked to SA- and JA/ET-related defence programs and to antiviral responses, which is consistent with the reported viral targeting of MED16. Finally, MED16 function appears to be sensitive to dosage, and the *MED16* locus may add further regulatory layers, including circular RNAs reported in maize.

### 5.2. Conservation of MED16 Function Beyond That in Arabidopsis

MED16 is a conserved subunit of the Mediator complex in plants, and homologs are present in many crop and non-model species. In *Arabidopsis*, studies on MED16/SFR6 link it to stress-responsive transcription, where it appears to act after stress-related transcription factors bind DNA and help support effective Mediator and RNA polymerase II functions at target genes. In non-model plants, most evidence thus far comes from genome annotation and transcriptome datasets. These data suggest that MED16 orthologs are expressed in many tissues and often change with abiotic stress and hormone cues, suggesting a broad role in transcriptional regulation. In crops such as rice, changes in *OsMED16* levels have been associated with strong developmental defects and defence-related transcriptional shifts, which is consistent with a dosage-sensitive role that may be shared across species. MED16 also seems to be associated with hormone-regulated transcription through Mediator, but direct mechanistic tests in crops and woody plants are still limited. In woody perennials and other non-model species, functional data remain scarce, yet expression patterns are often consistent with roles in growth and environmental acclimation.

Overall, the available evidence supports the conservation of *MED16* at the level of core transcriptional control, whereas species-specific outcomes likely reflect differences in upstream regulatory networks; more functional and mechanistic work in crops and woody plants is needed to link these patterns to traits and applications.

### 5.3. Controversies and Limitations in Understanding MED16 Function

Despite clear evidence that MED16 is involved in multiple stress- and hormone-responsive transcriptional programs, its role in plant immunity is still difficult to define in a single, consistent way. A key reason is that immune outputs are strongly context-dependent: SA- and JA/ethylene-related defence programs can interact in antagonistic or conditional ways, so MED16-associated phenotypes may differ across pathogen types, tissues, and experimental settings without necessarily implying opposite molecular functions. Current data also suggest that MED16 does not function as a universal activator or repressor. Instead, it appears to modulate transcription in a transcription factor- and pathway-dependent manner, which makes its immune role highly sensitive to the specific regulatory environment.

Another major limitation is the experimental design used in much of the literature. Many studies have examined MED16 under single stresses or single signalling pathways in isolation, which limits direct comparisons across pathogens and obscures how MED16 behaves when plants face combined stresses. In addition, mechanistic insights are dominated by work in *Arabidopsis*, whereas evidence from crops and other species is still limited. As a result, how broadly specific MED16-dependent immune features can be generalized across plants remains unclear. Overall, the main “controversies” associated with the role of MED16 in immunity likely reflect signalling crosstalk, differences in experimental context, and uneven species coverage rather than strong evidence that MED16 plays fundamentally opposing roles.

### 5.4. Future Directions

Future work on MED16 can be summarized into three connected directions. First, more studies are needed in crop and other non-model plants because most of the related mechanistic knowledge still comes from *Arabidopsis*. The results in crops suggest that MED16 function can be dosage-sensitive; thus, practical applications will likely require fine control of expression or activity rather than simple knockout or strong overexpression. Second, MED16 should be studied in the context of the entire Mediator complex. MED16 acts with other subunits in a cooperative or competitive way, but how these relationships change across tissues and conditions and how they shape RNA polymerase II engagement and transcriptional output are still unclear. Third, the physical basis of the interactions between MED16 and transcription factors remains unclear. Structural and mechanistic work, including high-resolution approaches and improved modeling, will be important for explaining how MED16 achieves context-specific integration of upstream signals. In addition, circular RNAs produced from *MED16* loci, as reported in maize, point to a possible extra regulatory layer that should be tested across species. Overall, filling these gaps will help connect MED16-centered mechanisms to plant traits under realistic environments and clarify how this Mediator subunit helps balance growth with stress adaptation.

## Figures and Tables

**Table 1:** Directly interacting transcription factors of MED16 across species and their associated signalling contexts and representative output genes.

Directly Interacting TF (Protein)	Species	Signalling Pathway/Biological Process	Functional Consequence	Representative Output Genes	Reference
DEL1	*A. thaliana*	Cell cycle control; endoreduplication	Transcriptional repression	*CCS52A2*	[12]
ABI5	*A. thaliana*	ABA signalling	Transcriptional activation	*EM1*, *EM6*	[16]
WRKY33	*A. thaliana*	JA/ET-mediated defence against necrotrophic pathogens	Transcriptional activation via Pol II recruitment	*PDF1.2*, *ORA59*	[38]
STOP1	*A. thaliana*	Phosphate starvation response; root growth inhibition	Transcriptional activation and Pol II recruitment	*ALMT1*	[11]
OsARF10	*Oryza sativa*	Auxin signalling; crown root initiation	Transcriptional activation	*OsCrl5*	[29]
BnWRKY33	*Brassica napus*	Defence signalling during *Sclerotinia sclerotiorum* infection	Strengthened defence program output	Not specified in the text	[41]

## Data Availability

No new data were created or analyzed in this study. Data sharing is not applicable to this article.
